# Supplementary material for: Mechanisms governing the pioneering and redistribution capabilities of the non-classical pioneer PU.1
Source: Nat Commun. 2020 Jan 21;11:402. doi: 10.1038/s41467-019-13960-2 (PMC6972792; doi:10.1038/s41467-019-13960-2)
Supplement: Supplementary file 7 — Source data [file 41467_2019_13960_MOESM7_ESM.zip › Source_Data/FigureS2/FigureS2B_MotifScanOutput/KmeansCluster13/homerResults/motif17.similar.html]

motif17

## Information for motif17

T
C
G
A
A
T
G
C
C
G
T
A
A
C
G
T
G
T
A
C
C
G
T
A
T
G
C
A
C
T
G
A
C
T
A
G
T
A
G
C
  
Reverse Opposite:  

A
T
C
G
A
G
T
C
A
G
C
T
A
C
G
T
A
C
G
T
A
C
T
G
G
T
C
A
G
C
A
T
T
A
C
G
A
G
C
T
  

|  |  |
| --- | --- |
| p-value: | 1e-27 |
| log p-value: | -6.317e+01 |
| Information Content per bp: | 1.647 |
| Number of Target Sequences with motif | 226.0 |
| Percentage of Target Sequences with motif | 13.36% |
| Number of Background Sequences with motif | 2670.7 |
| Percentage of Background Sequences with motif | 5.92% |
| Average Position of motif in Targets | 102.1 +/- 53.4bp |
| Average Position of motif in Background | 101.3 +/- 63.6bp |
| Strand Bias (log2 ratio + to - strand density) | 0.2 |
| Multiplicity (# of sites on avg that occur together) | 1.08 |
| Motif File: | file (matrix) reverse opposite |

### Similar de novo motifs found

|  |  |  |  |  |  |  |  |
| --- | --- | --- | --- | --- | --- | --- | --- |
| Rank | Match Score | Redundant Motif | P-value | log P-value | % of Targets | % of Background | Motif file |
| 1 | 0.765 | G T A C G C T A T A G C G T C A A C G T T A G C G C T A T C A G C T G A C T A G T A C G | 1e-26 | -62.066914 | 16.73% | 8.34% | motif file (matrix) |
| 2 | 0.878 | A G T C C G A T A G C T G A C T A C T G G C T A C G T A A T C G | 1e-21 | -49.688599 | 17.49% | 9.68% | motif file (matrix) |
| 3 | 0.895 | T G A C G C A T A G C T G A C T A T C G C G T A C G T A A T C G A G C T | 1e-18 | -41.525135 | 4.96% | 1.55% | motif file (matrix) |
| 4 | 0.846 | G T A C A C G T A C G T A C G T A C T G C G T A C G A T A G T C C G A T | 1e-14 | -34.011753 | 3.49% | 0.97% | motif file (matrix) |
